# Chitosan-Based Composite Films Reinforced with Zein–Inulin–Thyme Essential Oil Pickering Emulsion for Enhanced Structural Integrity and Preservation Capacity

**DOI:** 10.3390/foods15030484

**Published:** 2026-01-31

**Authors:** Liufeng Wang, Hongxin Xue, Yujie Ling, Xinping Zhong, Kuntai Li, Qiuming Zheng, Xiaoqing Chen, Xinyi He, Minghui Tan

**Affiliations:** College of Food Science and Technology, Guangdong Ocean University, Guangdong Provincial Key Laboratory of Aquatic Product Processing and Safety, Guangdong Provincial Engineering Technology Research Center of Seafood, Guangdong Province Engineering Laboratory for Marine Biological Products, Key Laboratory of Advanced Processing of Aquatic Product of Guangdong Higher Education Institution, Zhanjiang 524088, China; 13979805895@163.com (L.W.); xuehongxin666@163.com (H.X.); lyj2232206067@163.com (Y.L.); 13760990604@163.com (X.Z.); 14775539198@163.com (Q.Z.); 13415778249@163.com (X.C.); 19727687140@163.com (X.H.)

**Keywords:** Pickering emulsion, thyme essential oil, sustained release, preservation

## Abstract

Herein, zein–inulin-stabilized thyme essential oil (TEO) Pickering emulsions were prepared via ultrasonication. The addition of inulin (0.12–0.5%) enhanced emulsion stability and antibacterial activity, with particle sizes ranging from 73.7 to 789.8 nm. Chitosan (CS) composite films were then fabricated using different TEO loading methods. Films incorporating Pickering emulsions exhibited denser and smoother structures due to hydrogen bonding between the emulsion and chitosan matrix, while electrostatic interactions between zein and inulin enabled effective TEO encapsulation. Compared to the pure CS film, the Pickering emulsion active films exhibited improved thermal stability, with a maximum decomposition temperature of 260 °C, blocked up to 82.22% of UV light in the UVA region (320–400 nm), displayed increased hydrophobicity (maximum water contact angle of 75.70°), and showed the strongest scavenging activity toward both DPPH (93.27%) and ABTS (98.42%). Moreover, these films effectively reduced weight loss, minimized firmness decline, suppressed pH increase, and inhibited microbial growth, thereby delaying blueberry spoilage. Based on the appearance and total soluble solids content of blueberries, the chitosan Pickering emulsion (containing 0.25% inulin) film (type VI) presented the best preservation performance among the eight tested films. This study highlights the potential of chitosan-based Pickering emulsion active films for food packaging applications.

## 1. Introduction

With the continuous improvement of living standards, consumers are placing increasing emphasis on the freshness and safety of food. The growing reliance on non-biodegradable, petroleum-based plastics in food packaging poses serious environmental and safety challenges, driving the search for sustainable alternatives [1]. Biopolymers such as chitosan (CS) have emerged as promising candidates due to their biocompatibility, biodegradability, and favorable film-forming properties [2,3,4,5]. However, pure chitosan films exhibit inherent limitations, including poor UV barrier properties and insufficient antioxidant and antibacterial activities, which restrict their effectiveness in preventing food spoilage caused by oxidation and microbial growth [6,7,8].

To enhance functionality, the incorporation of bioactive compounds like plant essential oils into chitosan matrices has been widely explored [3]. Liang et al. developed an edible composite film from chitosan and oxidized fucoidan, incorporating cinnamaldehyde to achieve pH-responsive release behavior and significantly extend the shelf life of litchi [9]. Qian et al. fabricated a chitosan-dialdehyde cellulose composite film that incorporated both proanthocyanidins and carvacrol essential oils, resulting in effective inhibition of colony growth on strawberry surfaces [10]. Thyme essential oil (TEO) is frequently utilized in food packaging materials owing to its potent antibacterial and antioxidant properties [11,12,13]. However, its high volatility, poor water solubility, and instability limit its practical application [14]. Emulsification represents a viable strategy to address these issues. While conventional surfactants (e.g., Tween 80) can improve oil dispersion [15], they raise potential health concerns [16]. Pickering emulsions (PEs), commonly described as biopolymer-stabilized emulsions, offer a robust and sustained platform for the delivery of bioactive compounds. Compared to surfactants, PEs exhibit distinct advantages such as a thicker particle-stabilized interfacial layer, which contributes to enhanced protection and controlled release of encapsulated bioactive compounds, along with improved safety profiles, biodegradability, and environmental compatibility [17]. Yang et al. prepared a carboxylated cellulose nanocrysta-stabilized oregano essential oil Pickering emulsion (COPE), which effectively improved the bioactive, mechanical, and hydrophobic properties of the pectin films [18]. Fan et al. incorporated a cinnamon essential oil-loaded Pickering emulsion into chitosan/gelatin films, which improved the films’ antimicrobial and controlled-release properties [19].

While the individual and combined applications of chitosan films, Pickering emulsions, essential oils, and their role in fruit preservation are well-documented, a critical gap remains in developing edible films that synergistically combine superior physical stability with controlled antimicrobial release. Existing protein–polysaccharide-based Pickering systems, such as those utilizing chitosan or soy protein isolate, often face challenges related to interfacial rigidity, long-term coalescence stability, or require complex chemical modifications for optimal performance. To address this, the present study introduces a novel, all-natural Pickering emulsion system stabilized by a zein–inulin complex. The system leverages the unique molecular structure and self-assembly behavior of zein, which synergistically enhances emulsion stability through the formation of nanocomplexes with polysaccharides via electrostatic, hydrophobic, and hydrogen-bonding interactions [20,21,22,23,24]. Inulin, a linear fructan primarily composed of D-fructose units linked by β-(2→1)-glycosidic bonds, significantly enhances the emulsifying activity index (EAI) and emulsifying stability index (ESI) of emulsions [25,26], making it particularly valuable for developing emulsion-based active packaging materials. The novelty of this approach lies in its dual stabilization mechanism: hydrophobic zein nanoparticles spontaneously adsorb at the oil–water interface to form a primary particulate barrier, while hydrophilic inulin simultaneously constructs a viscoelastic network in the aqueous phase, thereby suppressing droplet coalescence and phase separation. Subsequently, the emulsion was incorporated into a chitosan matrix to fabricate active films. The physicochemical and functional properties of these films were systematically compared with those prepared using other methods for TEO loading. Finally, the efficacy of the optimized film in preserving blueberries was assessed. This work is expected to provide a valuable reference for the application of chitosan-based Pickering emulsion active films in food packaging.

## 2. Materials and Methods

### 2.1. Materials and Chemicals

Zein (purity ≥ 95%), chitosan (deacetylation degree > 95%), inulin (average degree of polymerization: 12), TEO, ethanol, glucose, and acetic acid were purchased from Macklin Biochemical Co., Ltd. (Shanghai, China), and all of which were at least analytical pure. Fresh blueberries were obtained from Zhanjiang, China.

### 2.2. Preparation of Pickering Emulsions

A certain amount of zein powder was weighed and then dissolved in 75% (*v*/*v*) ethanol to prepare a 1% zein solution. A measured quantity of inulin powder was dissolved in distilled water to prepare solutions at concentrations of 0%, 0.12%, 0.25%, 0.37%, and 0.5%, which were then stored at 4 °C for further use.

Under magnetic stirring, 30 mL of different concentrations of inulin solution (0%, 0.12%, 0.25%, 0.37%, and 0.5%) was gradually added to 10 mL of 1% (*v*/*v*) zein solution and stirred continuously for 30 min until a homogeneous mixture was obtained. The ethanol was subsequently removed by rotary evaporation (50 °C, 0.1 MPa), and the resulting concentrate was reconstituted to its original pre-evaporation volume with deionized water. The composite solution was initially sonicated for 5 min at 68% power using an ultrasonic cell crusher. Subsequently, 5 mL of TEO was introduced dropwise under continuous sonication, and the mixture was further processed for 15 min. Following pH adjustment to 9.2–9.5, a final 5 min sonication step yielded a milky-white Pickering emulsion.

### 2.3. Characterization of Pickering Emulsions

#### 2.3.1. Particle Size

The particle size of TEO-loaded Pickering emulsions was analyzed by a Laser Particle Size Analyzer (Mastersizer 2000, Malvern, UK). Specifically, the prepared Pickering emulsions were firstly diluted 10 times with ultrapure water, then determined at 25 °C and equilibrium time of 60 s [27].

#### 2.3.2. Microstructure

Each sample was deposited onto a glass slide, covered with a coverslip, and observed under an optical microscope at 1000× magnification to analyze microstructure.

#### 2.3.3. Emulsification Index (EI)

The emulsification index (EI), which directly indicates the interfacial stability of particle-stabilized Pickering emulsions, was determined according to the method of Shao et al. [28] with minor modifications. Briefly, 15 mL of the emulsion was transferred into a sealed glass vial and stored at 25 ± 1 °C for 7 days. The height of the emulsion layer (He) was measured every two days and compared to the total liquid height (Ht) for the calculation of the emulsification index. The EI was then calculated using the following equation:

(1) $$\mathrm{EI}\;(\%)\;=\;\frac{\mathrm{He}}{\mathrm{Ht}}\times 100\%$$

#### 2.3.4. Antibacterial Effect

The antibacterial activity of the Pickering emulsions was assessed against *Escherichia coli* and *Staphylococcus aureus* using the Oxford cup method [29]. Specifically, the microorganisms were first activated by culturing in an agar medium. Subsequently, bacterial suspensions of each strain were adjusted to 10^6^ CFU/mL and uniformly coated onto agar plates. After placing Oxford cups onto the plates, 150 μL of the emulsion was added to each cup. The assemblies were incubated at 37 °C for 24 h, and the antibacterial activity was quantified based on the observed inhibition zone diameter.

### 2.4. Preparation of Chitosan Pickering Emulsions Composite Films

**Figure 1:**
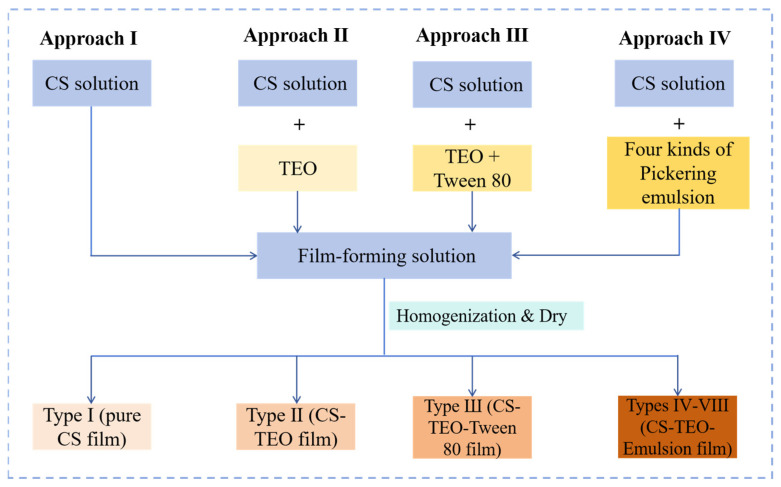
Preparation strategies of the four types of CS-based active films loaded with TEO.

Chitosan (1 g) was dissolved in 100 mL of an aqueous acetic acid solution (1% *v*/*v*) under constant magnetic stirring at 800 rpm for 2 h at 25 °C, resulting in a homogeneous solution with a pH of 5.0. Subsequently, glycerol was introduced into the solution at a concentration of 1% (*v*/*v*) under continuous stirring to ensure thorough mixing. The composite films were then fabricated according to the method described by Liu et al. [10], with minor modifications as illustrated in Figure 1. The films were prepared according to four different approaches. First, 50 mL of chitosan solution was used to fabricate a control film (type I). Subsequently, 1.7% (*v*/*v*) TEO was incorporated to yield type II film, and both 1.7% (*v*/*v*) TEO and 2% (*w*/*v*) Tween 80 were incorporated to yield type III film. For the type IV–VIII films, 7 mL of previously prepared Pickering emulsions (P-0%, P-0.12%, P-0.25%, P-0.37%, and P-0.5%) was slowly added to 43 mL of chitosan solution under magnetic stirring. The mixture was stirred continuously for 20 min to form a uniform active film solution. Finally, all film solutions (types I–VIII) were cast into culture dishes and dried in an oven at 35 °C for 24 h. The dried films were then peeled off and stored for further use.

### 2.5. Characterization of Prepared Films

#### 2.5.1. Scanning Electron Microscope (SEM)

The film samples were mounted on specimen stubs using conductive adhesive and subsequently sputter-coated with a thin gold layer for 2 min to enhance conductivity. The surface morphology of the films was then imaged using a field-emission scanning electron microscopy (FE-SEM, JSM-6700F, JEOL, Tokyo, Japan), with sequential magnifications (100×, 500×, 1000×, 2000×).

#### 2.5.2. Fourier Transform Infrared (FTIR) Spectroscopy

The FTIR spectrum of the prepared film was tested using an FTIR spectrometer (PE Frontier, Waltham, MA, USA). The dried film sample was placed on a diamond crystal, and the FTIR spectrum of the sample was measured in the range of 4000–400 cm^−1^ with a resolution of 4 cm^−1^ [30].

#### 2.5.3. X-Ray Diffraction (XRD)

The crystal structure of the film was analyzed by a Bruker AXS diffractometer (Karlsruhe, Germany) with Cu Kα radiation at 40 kV and 40 mA. Scans were recorded in the 2θ range of 5–80° with a scan rate of 5°/min [31].

#### 2.5.4. Thermogravimetric Analysis (TGA)

The thermal stability of the prepared films was analyzed using a thermogravimetric-differential thermal analyzer (TG-DTA 6300, Tokyo, Japan). The measurement was conducted with approximately 5 mg of the film sample sealed in an aluminum crucible. The sample was heated from 25 to 800 °C with a heating rate of 10 °C/min under a nitrogen atmosphere, with the gas flow rate maintained at 20 mL/min.

#### 2.5.5. Light Transmittance

The light transmittance of the films was determined following the procedure described by Khan et al. [32]. Rectangular strips (4 cm × 1 cm) were cut and placed in a colorimetric cuvette. An empty cuvette was used as the blank, and the transmittance of the samples was measured across the wavelength range of 200 to 800 nm using a UV–visible spectrophotometer (UV-2802PCS, UNICO, Shanghai, China).

#### 2.5.6. Water Vapor Permeability (WVP)

The WVP of the composite films was determined according to a previously described method [33]. A 4 cm × 4 cm film section was placed (ZCS side up) over the opening of a bottle containing 3 g anhydrous CaCl_2_ and secured with a rubber band. The bottle was then sealed in a desiccator with a saturated NaCl solution, maintaining a relative humidity of 75 ± 2%. The weight of the permeation bottle was recorded at 24-h intervals. The WVP of the composite film was determined according to Equation (2).

(2) WVP = (Δm × d)/(A × Δt × ΔP)

where Δm (g) denotes the change in bottle mass, d (m) is the thickness of the composite film, Δt (s) corresponds to the experimental duration, A (m^2^) represents the effective area of the composite film covering the weighing bottle mouth, and ΔP (Pa) is the difference in water vapor partial pressure between the interior of the bottle and the saturated sodium chloride solution maintained at 25 °C.

#### 2.5.7. Mechanical Properties

The mechanical properties, including tensile strength (TS) and elongation at break (EB), were evaluated according to the method described by Giteru et al. [34] with minor modifications. Specifically, rectangular strips (2 cm × 6 cm) were cut from the composite films and subjected to testing on an electronic universal testing machine (ZwickRoell Z030TE, Ulm, Germany).

#### 2.5.8. Thickness

The film thickness was measured at six random locations using a digital micrometer (293-340-30, Mitutoyo, Yokohama, Japan).

#### 2.5.9. Moisture Content (MC), Swelling Degree (SD) and Water Solubility (WS)

The film was cut into 3 cm × 3 cm squares. The initial mass (M_1_) of the sample was recorded, after which it was dried to a constant weight, and its mass was recorded again (M_2_). The MC of the prepared films was then calculated using Equation (3):

(3) $$\mathrm{MC}(\%)=\frac{M_{1}-M_{2}}{M_{1}}\times 100$$

The dried film sample was immersed in distilled water for 24 h to determine its swelling properties. The mass of the swollen film (M_3_) was measured after carefully removing surface water. The sample was then re-dried to a constant weight, yielding the final mass (M_4_). The swelling degree (SD) and water solubility (WS) were calculated according to Equations (4) and (5), respectively.

(4) $$\mathrm{SD}\;(\%)=\frac{M_{3}-M_{2}}{M_{2}}\times 100$$

(5) $$\mathrm{WS}\;(\%)=\frac{M_{2}-M_{4}}{M_{2}}\times 100$$

#### 2.5.10. Contact Angle Analysis

The surface hydrophobicity of the prepared films was characterized by water contact angle (WCA) analysis performed on a contact angle analyzer (DSA 25, Germany). Following the sessile drop method [35], film samples were cut into 2 cm × 6 cm strips and equilibrated at ambient conditions before testing. A 5 μL droplet of ultrapure water was then dispensed onto the film surface, and the initial static contact angle was captured and analyzed.

#### 2.5.11. Antioxidant Activity

The DPPH radical scavenging activity of the prepared films was determined according to the method of Kalkan et al. [36]. Specifically, 50 mg of film sample was added to 4 mL of 100 μmol/L DPPH ethanol solution, mixed thoroughly, and reacted in the dark at room temperature for 30 min. Then, the absorbance at 517 nm was measured using a UV–visible spectrophotometer (UV-2802PCS, UNICO, China). The DPPH radical scavenging activity was calculated according to Formula (6):

(6) $$\mathrm{Scavenging}\;\mathrm{rate}\;(\%)\;=\;(1\;-\;\frac{A_{s}-A_{\mathrm{sb}}}{A_{c}-A_{\mathrm{cb}}})\times 100$$

where A_s_ represents the absorbance of the sample solution with DPPH, A_sb_ represents the absorbance of film samples with 50% ethanol, A_c_ represents the absorbance of DPPH solution with solvent, and A_cb_ represents the absorbance of 50% ethanol with solvent.

The ABTS radical scavenging activity of prepared films was determined according to the method of Xiao et al. [37]. The ABTS^+^· solution was prepared by combining equal volumes (10 mL each) of ABTS solution and potassium persulfate solution, followed by 12–16 h of dark incubation at room temperature. Prior to use, the ABTS^+^· working solution was diluted with phosphate-buffered saline (PBS, pH 7.4) to an absorbance of 0.70 ± 0.02 at 734 nm. Subsequently, 50 mg of the film sample was combined with 4 mL of the diluted ABTS^+^· solution. The mixture was incubated in the dark at room temperature for 6 min, after which its absorbance was measured at 734 nm. The ABTS radical scavenging activity was calculated using Formula (7):

(7) $$\mathrm{Scavenging}\;\mathrm{rate}\;(\%)\;=\;(1-\frac{A_{s}}{A_{c}})\times 100$$

where A_c_ represents the absorbance of the pure ABTS^+^· solution, and A_s_ represents the absorbance of the film samples and ABTS^+^· solution.

### 2.6. Application in Blueberry Preservation

Blueberries were selected for the preservation experiment based on uniform size and intact skins free from any physical damage. Prior to testing, all blueberries were rinsed in deionized water for 5 min and air-dried. The fruits were then randomly assigned to different groups and packaged with the prepared composite films. A set of unpackaged samples was designated as the blank control. All samples were stored under controlled conditions of 25 ± 1 °C and 50% relative humidity (RH) for a total of 7 days. Key quality indicators were evaluated at two-day intervals.

#### 2.6.1. Appearance Changes

The morphological changes in blueberries in different treatment groups were documented photographically throughout the storage period.

#### 2.6.2. Weight Loss Rate

The weight loss rate of blueberries during storage was measured by the gravimetric method and calculated by the following formula:

(8) $$\mathrm{Weight}\;\mathrm{loss}\;\mathrm{rate}\;(\%)\;=\;\frac{W_{0}-W_{n}}{W_{0}}$$

where W_0_ was the initial weight (g), and W_n_ was the weight (g) of blueberries during storage at day n.

#### 2.6.3. Hardness

A handheld hardness tester (GY-2, China) was used to measure the hardness of blueberries by recording the peak force from multiple locations on each fruit, with the average value used for subsequent analysis.

#### 2.6.4. pH, Reducing Sugar and Total Soluble Solids (TSS) Content

Ten grams of blueberry pulp was mixed with 20 mL of distilled water and homogenized using a high-speed shear machine at a speed of 5000 r/min for 3 min. After standing still, the pH value of the supernatant was measured using a pH meter. The reducing sugar content of blueberry fruits during storage was determined by the 3,5-dinitrosalicylic acid (DNS) colorimetric method. A handheld refractometer (DR122 ATC, UK) was used to determine the TSS content of blueberries [35]. Measurements were taken every two days, with each sample analyzed in triplicate.

#### 2.6.5. Microbial Counts

Each group of blueberry samples (six blueberries) was homogenized with 50 mL of sterile water under continuous stirring for 10 min. The resulting homogenate was serially diluted, and 1 mL of the appropriate dilution was mixed with molten agar medium. Following complete solidification, the plates were inverted and incubated under specific conditions: LB agar at 37 °C for 48 h for total plate count (TPC), and PDA agar at 25 °C for 5 days for yeasts and molds (Y&M). The TPC and Y&M counts were then calculated from the number of colonies and their corresponding dilution ratio, and expressed as colony-forming units per milliliter (CFU/mL) [38].

### 2.7. Statistical Analysis

Data were expressed as mean ± standard deviation, and were plotted using Origin 2021. The significant differences between means were determined by Duncan’s multiple range test (*p* < 0.05) using SPSS Statistical 26.0.

## 3. Results and Discussion

### 3.1. Characterization of Pickering Emulsions

#### 3.1.1. Appearance Changes

**Figure 2:**
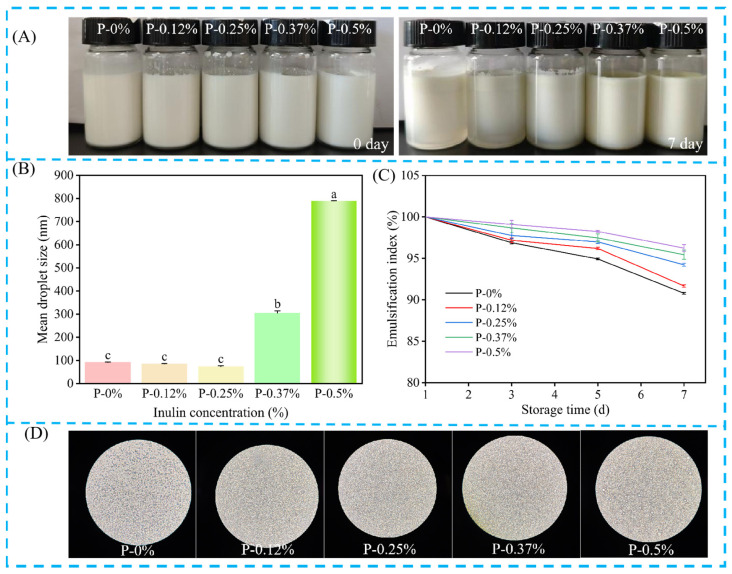
Effect of inulin concentration on the properties of Pickering emulsion: (**A**) Visual observation images of Pickering emulsion stored for 0 and 7 days, (**B**) Emulsification index (Different letters indicate significant differences (*p* < 0.05)), (**C**) Mean droplet size, (**D**) Optical micrograph of Pickering emulsion stored for 7 days.

As presented in Figure 2A, emulsions containing lower inulin concentrations (0%, 0.12%, and 0.25%) were subject to phase separation within 7 days of storage, a result of gravitational instability owing to the density contrast between phases [11]. Conversely, higher inulin concentrations (0.37% and 0.5%) conferred enhanced stability, with the corresponding emulsions showing no phase separation during the observation period.

#### 3.1.2. Particle Size

The particle size of Pickering emulsions critically determines their environmental stability. As shown in Figure 2B, the prepared Pickering emulsion had a nanometer-scale particle size, with an average range of 73.7 to 789.8 nm. The particle size of the emulsion initially decreased with increasing inulin content, reaching a minimum at 0.25%, and then showed a trend of gradual increase. This trend was consistent with the results of optical microscopy observations.

#### 3.1.3. EI Value

EI was an important index to reflect the stability of Pickering emulsion. As shown in Figure 2C, the EI value increased with the increase in inulin content, and the layering phenomenon of Pickering emulsion gradually weakened. After 7 days of storage, the EI varied significantly among formulations. The P-0% emulsion (control) showed the lowest EI (90.5%) and exhibited severe phase separation, whereas the P-0.5% Pickering emulsion achieved the highest EI (96%), indicating optimal stability. The observed improvement in emulsion stability may be attributed to the increased polysaccharide content, enhancing protein–polysaccharide complexation. This synergistic interaction promotes the formation of a robust interfacial layer around TEO droplets, thereby improving emulsification efficacy.

#### 3.1.4. Microstructure

As shown in Figure 2D, the images of 0%, 0.12%, 0.25%, 0.37% and 0.5% emulsions at 1000× of the optical microscope exhibited a spherical shape, which is a common characteristic of Pickering emulsions [39]. The emulsion without inulin exhibited the largest particle structure. In addition, the water-in-oil particle structure decreased with increasing inulin content, reaching a minimum at 0.25%. This likely resulted from the saturation of inulin-protein binding, leading to minimal oil phase encapsulation. Beyond this point, particle size increased with further inulin addition.

#### 3.1.5. Antibacterial Activity

As shown in Figure S1, the prepared Pickering emulsions demonstrated antibacterial activity against both types of bacteria, and the antibacterial activity against *S. aureus* and *E. coli* was concentration-dependent, varying significantly with different inulin loading levels. At inulin concentrations below 0.25%, the emulsions exhibited a stronger antibacterial effect against *E. coli* than against *S. aureus*. However, this trend reversed when the inulin concentration exceeded 0.25%. The differential antibacterial effect likely arose from an interaction between the emulsions’ charge and the distinct cell wall structures of the bacteria [40]. Low concentrations of inulin may disrupt the outer membrane of *E. coli* via electrostatic effects, while higher concentrations may target the peptidoglycan layer of *S. aureus* through enhanced binding or pore formation.

### 3.2. Characterization of Pickering Emulsion Active Films

#### 3.2.1. SEM

**Figure 3:**
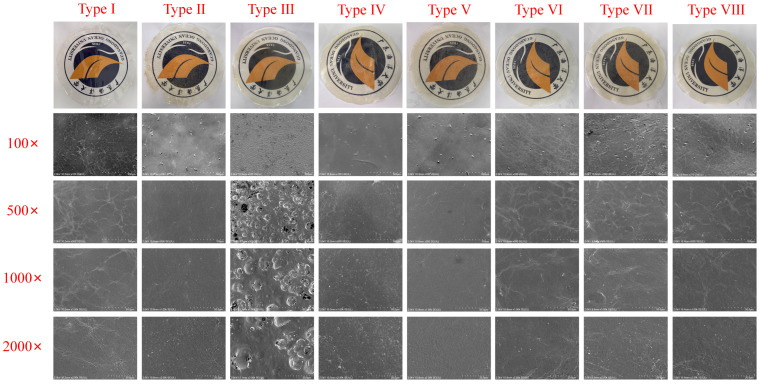
The microstructure of the prepared active films.

The surface morphology of the active film was observed by SEM, and varied significantly (Figure 3). The pure CS film (Type I) exhibited a smooth, void-free surface. While the type II film showed minor bubble formation, its structural integrity was largely maintained. Notably, the incorporation of Tween 80 and TEO (Type III) led to the formation of extensive bubble holes, which severely compromised the surface smoothness and sealing quality. In the Pickering emulsion films, a structural network was formed between the chitosan matrix and the zein–inulin particles, primarily through hydrogen bonding. Generally, all active films incorporating the Pickering emulsion exhibited smooth and dense structures without obvious cracks or voids, indicating that good compatibility was achieved between the chitosan matrix and the emulsion [41]. It is worth noting that examining film cross-sections would help clarify the distribution of Pickering emulsion nanoparticles and their influence on pore structure [42], which remains an important direction for subsequent investigation.

#### 3.2.2. FTIR

**Figure 4:**
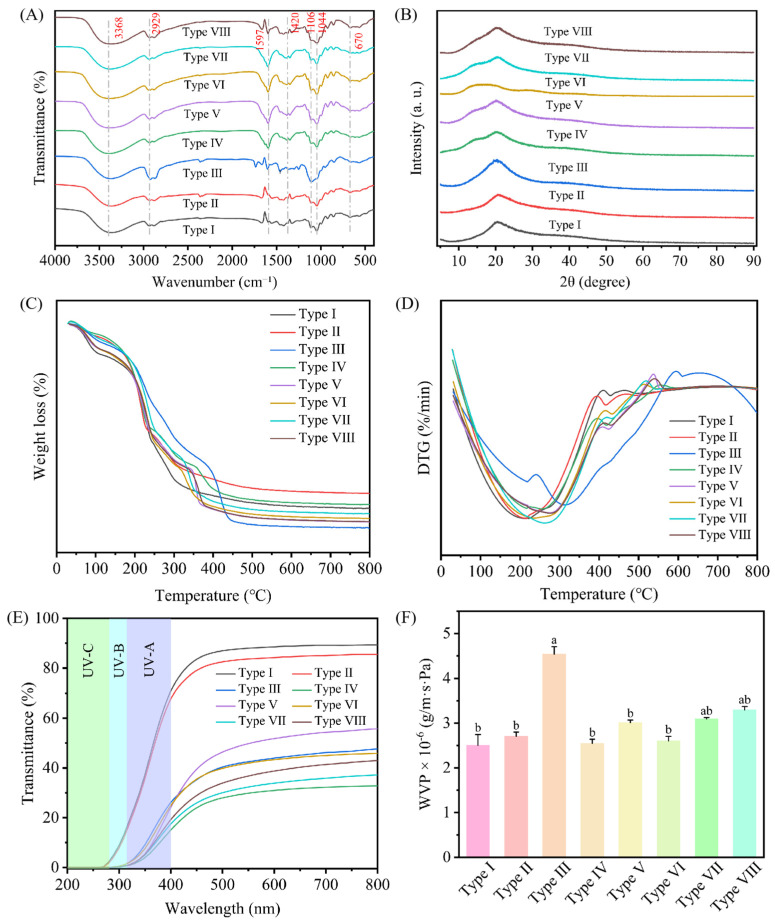
FTIR spectra (**A**), XRD (**B**), TG (**C**), DTG (**D**), light transmission characteristics (**E**) and water vapor permeability (**F**) of the active films. Distinct letters indicate significant differences (*p* < 0.05).

FTIR was performed to analyze the interactions between different components in the active film. As shown in Figure 4A, all prepared films exhibited similar infrared spectral characteristics. The peaks at 3368 and 2929 cm^−1^ were characteristic absorption peaks of O-H and C-H in chitosan. The peaks at 1420, 1044, and 670 cm^−1^ were attributed to the C=C stretching vibration, C-C stretching vibration, and molecular skeleton vibration of the aromatic ring, respectively. Compared to the pure CS film (Type I), the Pickering emulsion films exhibited a significantly enhanced absorption peak at 1597 cm^−1^. This enhancement was likely attributable to the bending vibration of protonated amino groups, suggesting their involvement in the electrostatic interaction between protein and polysaccharide within the Pickering emulsion. In addition, the slight changes in the absorption peak at 1420 cm^−1^ indicated the interaction between chitosan and inulin, leading to the formation of hydrogen bonds [43].

#### 3.2.3. XRD

The crystallization properties of composite films were determined using an X-ray diffractometer. As shown in Figure 4B, all films showed a characteristic diffraction peak at 2θ ≈ 20°. An obvious enhancement in this peak’s intensity was observed for the Pickering emulsion films compared to the pure CS film (Type I), indicating that the addition of the Pickering emulsion promoted crystallinity by facilitating a more ordered crystal structure. Furthermore, the appearance of a diffraction peak at 2θ = 14–15° exclusively in the Pickering emulsion films can be interpreted as evidence for the intermolecular interactions between the zein and chitosan [44].

#### 3.2.4. Thermal Properties

The thermal stability of the films was characterized by employing thermogravimetric (TG) and derivative thermogravimetric (DTG) analysis. As illustrated in Figure 4C, the film exhibited a gradual weight loss with increasing temperature, characterized by four distinct stages of thermal decomposition. The initial weight loss around 100 °C is attributed to water evaporation. The subsequent stage, observed between 100 and 200 °C, is likely due to the degradation of glycerol. The third stage (200–300 °C) corresponds to chitosan degradation, while the final stage (300–450 °C) is associated with the breakdown of the emulsion. As evidenced by the DTG curves in Figure 4D, the maximum decomposition temperature (T_max_) for both the pure CS film and the CS-TEO film was approximately 210 °C. In contrast, the T_max_ for the Pickering emulsion composite films increased notably to over 260 °C. This obvious shift demonstrates that incorporating the Pickering emulsion effectively enhanced the thermal stability of the chitosan-based film. The FTIR and XRD analyses collectively demonstrate that the introduction of the Pickering emulsion facilitates the formation of an extensive hydrogen-bonding network among the film components. This enhanced intermolecular interaction not only improves the structural ordering and crystallinity of the composite (as evidenced by XRD) but also contributes to the observed increase in thermal stability.

#### 3.2.5. Optical Transmittance

Ultraviolet (UV) radiation is a major factor contributing to the oxidation of unsaturated fatty acids [45]. Therefore, packaging materials generally need to possess a certain degree of UV-blocking properties. As demonstrated in Figure 4E, all tested films exhibited excellent barrier performance against UVC radiation (200–280 nm), showing consistently low light transmittance across this spectral range. While type I and II films showed UVB (280–320 nm) transmittance values of 18.20% and 17.06%, respectively, the values for types III-VIII films were significantly lower, remaining below 5%. Specifically, in the UVA region (320–400 nm), films IV-VIII effectively blocked up to 82.22% of the UV light, a significantly higher blocking capacity than that of films I and II. The reduction in film transparency may be explained by two factors. First, the incorporation of the Pickering emulsion may have introduced fine particles into the film matrix pores, thereby affecting light transmittance [46]. Second, the light-scattering properties of the essential oils, as reported by Chen et al. [47], further contributed to the reduction in film transparency.

#### 3.2.6. Water Vapor Permeability (WVP)

As shown in Figure 4F, the elevated WVP observed in the type III (CS-TEO-Tween 80) film is attributable to the surfactant nature of Tween 80. Its ability to lower surface tension and improve wettability likely contributes to this enhanced permeability. This observation aligns with the results of SEM image, which reveals a porous surface morphology characterized by numerous pores and incomplete fusion, accounting for the compromised barrier efficacy. Notably, among the Pickering emulsion films, types IV and VII demonstrated obviously lower WVP compared to the pure CS film. This enhanced barrier property can be attributed to the denser network formed through extensive hydrogen bonding between the zein–inulin complex and the chitosan matrix, as evidenced by FTIR and XRD analyses, which effectively impedes the diffusion of water vapor molecules [48]. However, the WVP of our films remains higher than that reported for high-barrier chitosan-based composites by Tang et al. (ranging from 1.88 × 10^−8^ to 5.04 × 10^−8^ g/(m·s·Pa)) [49], indicating clear potential for further optimization of the formulation or processing to enhance the water vapor barrier.

#### 3.2.7. Mechanical Properties and Thickness

**Figure 5:**
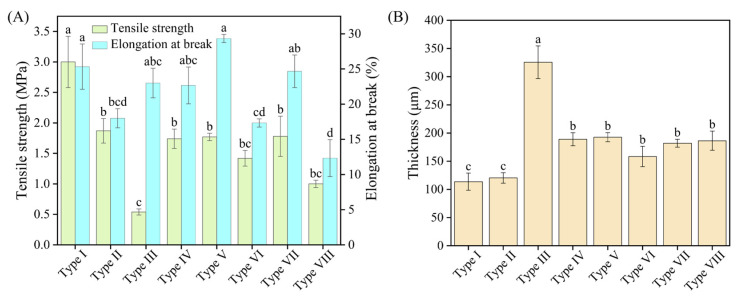
Tensile strength (TS) and elongation at break (TB) (**A**), thickness (**B**) of the active films. Distinct letters indicate significant differences (*p* < 0.05).

The tensile strength (TS) and elongation at break (EB) were determined to characterize the mechanical properties of the prepared films. As shown in Figure 5A, the type I (pure CS) film demonstrated significantly higher TS and EB values than the other films, indicating its superior mechanical properties. Although the type II (CS-TEO) film exhibited the second-highest TS, its ductility was inferior. The type III (CS-TEO-Tween 80) film demonstrated the lowest TS value among all tested samples. Among the Pickering emulsion films, type V was characterized by high TS and EB. Compared with the pure CS film, the addition of the Pickering emulsion decreases the tensile strength of the CS composite film to some extent, which may be attributed to changes in the microstructure (e.g., aggregates) induced by the emulsion. Nevertheless, the prepared Pickering emulsion film still shows potential for application in the field of food preservation.

The thickness of a film, governed by its fabrication protocol and drying regimen, is a critical factor affecting most of its key mechanical and physical properties. As displayed in Figure 5B, the thickness of the pure CS film (Type I) was 113.67 μm, and the addition of the different emulsifiers did alter the thickness of chitosan-based active films (*p* < 0.05). The type III film (CS-TEO-Tween 80) exhibited the greatest thickness among the eight tested films. This can be explained by the addition of Tween 80, which disrupted the intermolecular interactions between CS and TEO [45]. Consequently, the originally dense network structure broke down, resulting in the formation of a more loosely organized film matrix (also supported by SEM results). This looser matrix structure further contributed to the reduction in tensile strength, as illustrated in Figure 5A. The thickness of the Pickering emulsion films ranged from 158.33 to 192.67 μm, which was greater than that of the pure CS film but lower than that of the CS-TEO-Tween 80 film. This difference is likely attributable to potential hydrogen bonding and electrostatic interactions between CS and the nanoparticles in the Pickering emulsion, as supported by FTIR and XRD analysis. In summary, the incorporation of the Pickering emulsion resulted in a moderate decrease in tensile strength and a slight increase in film thickness, while also improving the elongation at break of the active film.

#### 3.2.8. Moisture Content (MC), Swelling Degree (SD), Water Solubility (WS) and Water Contact Angle (WCA)

The MC, SD, and WS of the film could affect its stability, thereby affecting its application [49]. As shown in Table 1, the reduction in MC, SD, and WS values observed for the TEO-composite film, compared to the pure CS film, suggests that the incorporation of TEO enhances the film’s hydrophobic character. It is speculated that this effect arises from effective intermolecular interactions between CS and TEO, which diminish the matrix’s capacity to absorb water. Moreover, the interaction between CS and the hydrophobic Pickering emulsion may have competitively replaced CS-water interactions [50].

The water contact angle (WCA), a key indicator of surface wettability, characterizes surfaces with values below 65° as hydrophilic [12]. As shown in Table 1, the pure CS film (Type I) exhibited a WCA of 40.90°, confirming its hydrophilic nature. In contrast, the Pickering emulsion films (Types IV-VIII) showed a gradual increase in WCA with rising inulin content, reaching a maximum value of 75.70° for type VII. This trend indicates that incorporating Pickering emulsion effectively enhanced the surface hydrophobicity of the composite films. Furthermore, the observed increase in WCA may also result from a reduction in surface-exposed hydroxyl groups, owing to the formation of hydrogen bonds between inulin and the film matrix [51].

#### 3.2.9. In Vitro Antioxidant Activity

**Figure 6:**
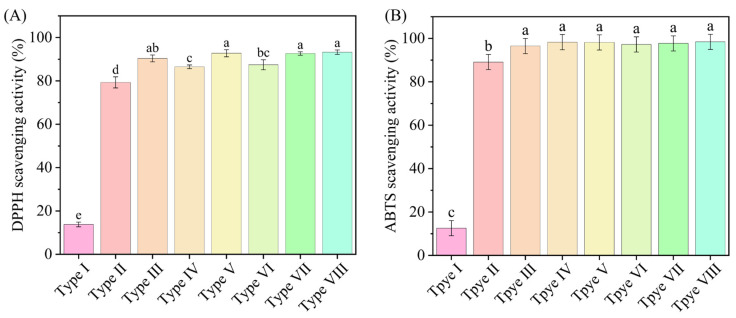
DPPH (**A**) and ABTS (**B**) radical scavenging activity of the active films. Distinct letters indicate significant differences (*p* < 0.05).

The antioxidant activity of TEO films prepared with different loading methods (Figure 6) substantiates the role of plant essential oils as natural antioxidants. It was observed that the type I film demonstrated the weakest scavenging activity against both DPPH and ABTS radicals. The addition of TEO led to a marked enhancement in the free radical scavenging rate, which is attributed to its inherent strong antioxidant properties. In addition, the films loaded with TEO using Tween 80 and the Pickering emulsion exhibited significantly enhanced oxidation resistance. Among them, the type VIII active film exhibited the highest scavenging activity (93.27% for DPPH and 98.42% for ABTS). These results demonstrated that encapsulating TEO within a Pickering emulsion is an effective approach for realizing sustained antioxidant release.

### 3.3. Application in Blueberry Preservation

#### 3.3.1. Appearance Changes

**Figure 7:**
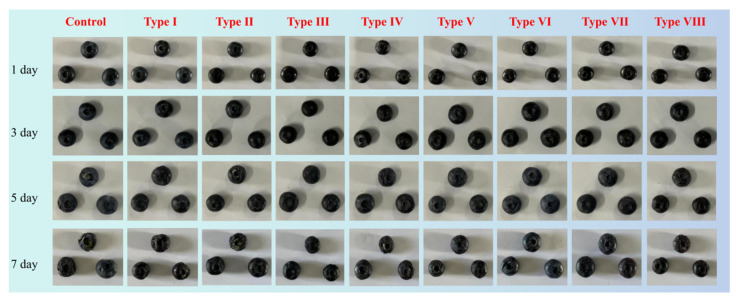
Appearance changes in blueberries stored for 7 days under different treatments.

In this study, blueberries were used to evaluate the freshness preservation ability of the prepared Pickering emulsion active film. As shown in Figure 7, the blueberries treated with Pickering emulsion composite film were shinier, which may be related to the antioxidant performance of TEO. With the extension of storage time, blueberries treated with uncoated (control), pure CS film (Type I), and CS-TEO film (Type II) began to rot on the third day, and began to mold on the fifth day. However, blueberries treated with Pickering emulsion composite film began to darken on the fifth day and showed signs of decay on the seventh day. Based on visual inspection, type VI film demonstrated superior preservation performance among the eight types films, followed by type VII film.

#### 3.3.2. Weight Loss Rate and Hardness

**Figure 8:**
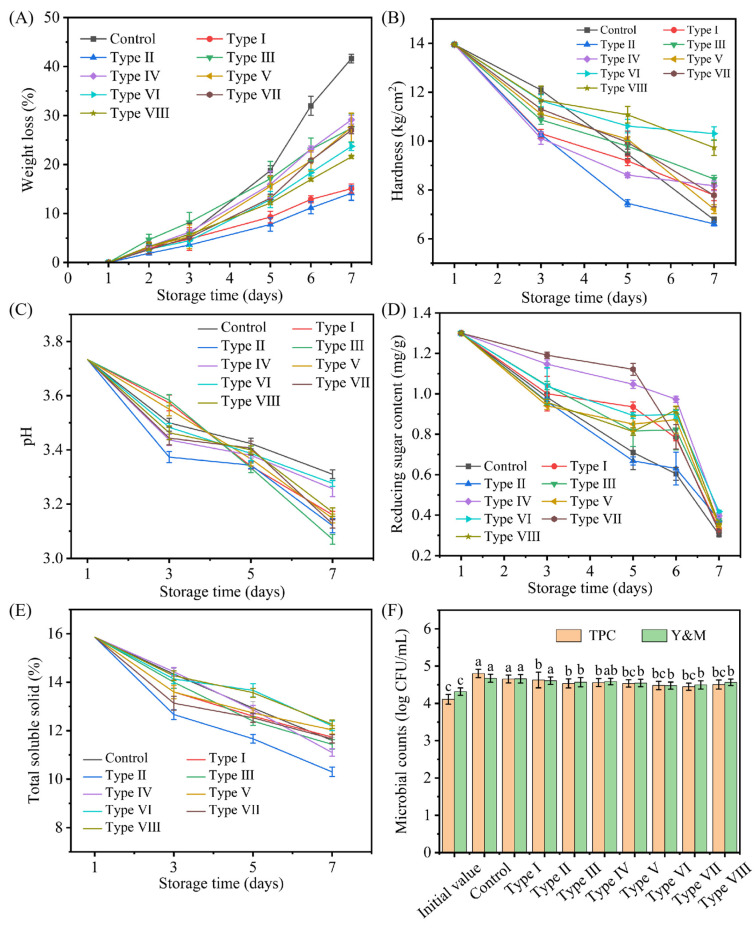
Effect of composite films on weight loss (**A**), hardness (**B**), pH (**C**), reducing sugar (**D**), TSS (**E**), and microbial counts (**F**) of blueberries during storage. Distinct letters indicate significant differences (*p* < 0.05).

The weight loss of fruits during storage is largely determined by their transpiration and respiration rates, as well as the gas barrier properties of the packaging film [52]. As shown in Figure 8A, the weight loss rate of blueberries exhibited an increasing trend with prolonged storage time. The samples maintained a constant mass for a period of one to three days at room temperature (25 ± 1 °C). A weight loss of 42% on day 3 indicated a marked quality deterioration in the control group (unwrapped blueberries), representing the most pronounced change among all samples. The Pickering emulsion film treatment reduced the weight loss rate of blueberries, with type VIII proving the most effective by yielding the lowest weight loss rate and the best preservation efficacy.

The compactness of blueberries was assessed by measuring the puncture hardness of their skin. The softening of blueberries resulted from water loss as well as enzymatic breakdown of cell wall polysaccharides [45]. As shown in Figure 8B, blueberries coated with type IV-VIII Pickering emulsion active films demonstrated superior hardness and a more gradual decline compared to other treatments. This indicates that the Pickering emulsion active film created a humid microenvironment, which reduced water loss. Furthermore, its potent antimicrobial activity inhibited microbial infection and fungal spore germination, thereby collectively delaying blueberry decay [53].

#### 3.3.3. pH, Reducing Sugar and TSS Content

The pH of blueberry pulp was measured, as it reflects the dynamics of organic acids throughout the storage period [42]. According to the literature, pH typically increases during storage due to the consumption of organic acids in respiration [54]. The observed decline in pH in our processed blueberries presents an apparent contradiction (Figure 8C), suggesting the involvement of an alternative, pH-lowering mechanism. In addition, the pH values of blueberries packaged with the CS-TEO-Tween 80 (Type III) film were consistently the lowest among all groups. Among the Pickering emulsion active film treatment groups, type IV and type VI exhibited the smallest reduction in pH compared to the other film formulations.

During fruit storage, the sensory and metabolic quality of blueberries, reflected by TSS, is largely determined by their primary components: soluble sugars and organic acids [55]. And the metabolic processes driving these changes involve the consumption of primary substrates like reducing sugars. As shown in Figure 8D and Figure 8E, blueberries packaged with the Pickering emulsion active film exhibited significantly higher soluble solids and glucose content compared to the control and non-Pickering film groups (Types I, II, III), respectively. On the seventh day, type VI showed the highest soluble solids content, while type VII showed the highest reducing sugar content of all components. These results collectively indicate that the Pickering emulsion active film delayed the degradation of soluble solids and reducing sugar, thereby better preserving the blueberries’ nutritional and sensory quality.

#### 3.3.4. Microbial Counts of Blueberry During Storage

The proliferation of microorganisms such as bacteria and fungi not only accelerates food spoilage but also presents substantial risks to human health. As shown in Figure 8F, type I (pure CS) film demonstrated the least effective antimicrobial activity among all tested formulations. A strong inhibitory effect against both bacteria and fungi was observed for the type III (CS-TEO-Tween 80) and Pickering emulsion active film, which showed a marked enhancement in antimicrobial efficacy over the control (*p* < 0.05). Moreover, the antimicrobial activity of the Pickering emulsion active film initially increased and then decreased with increasing inulin content. This pattern is likely due to the combined effect of the intrinsic antimicrobial property of TEO and the film’s structural regulation of internal moisture and oxygen [38].

## 4. Conclusions

This study systematically evaluated the effects of different TEO-loading methods on the properties of chitosan-based active films. SEM analysis indicated that incorporating Pickering emulsions yielded a denser film morphology. Integrating zein–inulin thyme essential oil Pickering emulsions into chitosan-based composite films improved thermal stability, enhanced UV barrier properties, increased hydrophobicity, and reinforced antioxidant activity. In blueberry preservation trials, films containing Pickering emulsions showed reduced weight loss, minimized firmness decline, suppressed pH increase, the lowest microbial counts, and a significantly delayed decay process. Together, these results demonstrate that TEO-loaded active films stabilized by Pickering emulsions represent a promising and viable strategy for food packaging applications. Nevertheless, further studies are necessary to assess the cytotoxicity, sensitization, and migration behavior of the film to fully evaluate its biosafety. Concurrently, future work should also extend its application to a wider range of high-value, perishable foods to verify its broad-spectrum preservation efficacy and practical applicability.

## Figures and Tables

**Table 1:** Analysis of MC, SD, WS and WCA of the active films.

Film Samples	MC (%)	SD (%)	WS (%)	WCA (°)
Type I	19.29 ± 1.24 ^a^	17.14 ± 2.86 ^a^	52.64 ± 2.32 ^a^	40.90 ± 3.21 ^c^
Type II	14.27 ± 0.63 ^c^	14.82 ± 1.44 ^b^	52.11 ± 7.11 ^a^	56.30 ± 3.04 ^b^
Type III	15.37 ± 2.19 ^bc^	9.98 ± 0.62 ^d^	38.68 ± 0.73 ^d^	15.13 ± 0.94 ^e^
Type IV	16.18 ± 0.45 ^bc^	7.75 ± 0.45 ^e^	47.63 ± 2.42 ^abc^	19.60 ± 0.46 ^e^
Type V	16.66 ± 0.29 ^bc^	11.32 ± 0.72 ^c^	45.18 ± 0.41 ^bc^	23.53 ± 3.61 ^d^
Type VI	16.44 ± 0.38 ^bc^	9.64 ± 0.81 ^d^	43.50 ± 0.58 ^bcd^	69.77 ± 3.52 ^a^
Type VII	17.57 ± 2.38 ^ab^	15.90 ± 1.90 ^ab^	41.41 ± 1.30 ^cde^	75.70 ± 0.26 ^a^
Type VIII	17.19 ± 0.69 ^ab^	16.79 ± 1.38 ^a^	36.60 ± 0.40 ^e^	56.97 ± 1.00 ^b^

Mean ± SD values followed by different superscript letters within a column denote statistically significant differences (*p* < 0.05).

## Data Availability

The original contributions presented in this study are included in the article/Supplementary Materials. Further inquiries can be directed to the corresponding authors.

## Supplementary Materials

The following supporting information can be downloaded at: https://www.mdpi.com/article/10.3390/foods15030484/s1, Figure S1: Antibacterial effect of Pickering emulsions. Distinct letters indicate significant differences (*p* < 0.05).

## References

[1] Tan W., Li Y., Guo X., Wei L., Duan J., Qi Z., Yuan Y., Chen Q., Guo Z. (2025). Enhanced ultraviolet barrier, antioxidant and antibacterial properties of chitosan-based films incorporated with caffeic acid–grafted inulin for strawberry preservation. Food Chem. X.

[2] Cao Y., Song Z., Ni W., Ma Y., Xin K., Yu Q., Zhang L. (2024). Composite nanoparticle-filled oxidized hydroxypropyl starch/carrageenan films: Robust, water-resistant, antibacterial, antioxidant and biodegradable properties. Food Hydrocoll..

[3] Fan Y., Ren J., Xiao X., Cao Y., Zou Y., Qi B., Luo X., Liu F. (2025). Recent advances in polysaccharide-based edible films/coatings for food preservation: Fabrication, characterization, and applications in packaging. Carbohydr. Polym..

[4] He B., Gao Y., Lou W., Wang J., Xin S., Ma K., Li L. (2025). Biodegradable food packaging films: Material source, mechanical and water vapor barrier properties, and improvement strategies—A comprehensive review. Food Res. Int..

[5] Kumar S., Shukla P., Das K., Katiyar V. (2025). Chitosan/water caltrop pericarp extract reinforced active edible film and its efficacy as strawberry coating for prolonging shelf life. Int. J. Biol. Macromol..

[6] Lee C.R., Lee S.J., Kim T.I., Chathuranga K., Lee J.S., Kim S., Kim M.H., Park W.H. (2025). Chitosan-gallic acid conjugate edible coating film for perishable fruits. Food Chem..

[7] Yu J., Xu S., Goksen G., Yi C., Shao P. (2023). Chitosan films plasticized with choline-based deep eutectic solvents: UV shielding, antioxidant, and antibacterial properties. Food Hydrocoll..

[8] Yuan Y., Tan W., Lin C., Zhang J., Li Q., Guo Z. (2023). Development of antioxidant chitosan-based films incorporated with chitooligosaccharide-caffeic acid conjugates. Food Hydrocoll..

[9] Liang F., Liu C., Geng J., Chen N., Lai W., Mo H., Liu K. (2024). Chitosan–fucoidan encapsulating cinnamaldehyde composite coating films: Preparation, pH-responsive release, antibacterial activity and preservation for litchi. Carbohydr. Polym..

[10] Qian L., Jia R., Zhao Q., Sun N., Yang J., Wen J., Li H., Yang J., Mo L., Gao W. (2025). Tough, antibacterial, and antioxidant chitosan-based composite films enhanced with proanthocyanidin and carvacrol essential oil for fruit preservation. Food Res. Int..

[11] Li H., Liu M., Han S., Hua S., Zhang H., Wang J., Xia N., Liu Y., Meng D. (2024). Edible chitosan-based Pickering emulsion coatings: Preparation, characteristics, and application in strawberry preservation. Int. J. Biol. Macromol..

[12] Liu Z., Lin D., Li N., Yang X. (2022). Characterization of konjac glucomannan-based active films loaded with thyme essential oil: Effects of loading approaches. Food Hydrocoll..

[13] Sun Y., Jia S., Hou Y., Cheng S., Tan M., Zhu B., Wang H. (2025). Novel thyme essential oil-loaded biodegradable emulsion film based on soybean lipophilic proteins for salmon preservation. Food Hydrocoll..

[14] Rout S., Tambe S., Deshmukh R.K., Mali S., Cruz J., Srivastav P.P., Amin P.D., Gaikwad K.K., Andrade E.H.A., Oliveira M.S. (2022). Recent trends in the application of essential oils: The next generation of food preservation and food packaging. Trends Food Sci. Technol..

[15] Song X., Zuo G., Chen F. (2018). Effect of essential oil and surfactant on the physical and antimicrobial properties of corn and wheat starch films. Int. J. Biol. Macromol..

[16] Cao T.L., Song K.B. (2019). Effects of gum karaya addition on the characteristics of loquat seed starch films containing oregano essential oil. Food Hydrocoll..

[17] Li J., Xu X., Chen Z., Wang T., Lu Z., Hu W., Wang L. (2018). Zein/gum Arabic nanoparticle-stabilized Pickering emulsion with thymol as an antibacterial delivery system. Carbohydr. Polym..

[18] Yang W., Zhang S., Hu Y., Fu Q., Cheng X., Li Y., Wu P., Li H., Ai S. (2024). Pectin-based film activated with carboxylated cellulose nanocrystals-stabilized oregano essential oil Pickering emulsion. Food Hydrocoll..

[19] Fan S., Wang D., Wen X., Li X., Fang F., Richel A., Xiao N., Fauconnier M.L., Hou C., Zhang D. (2023). Incorporation of cinnamon essential oil-loaded Pickering emulsion for improving antimicrobial properties and control release of chitosan/gelatin films. Food Hydrocoll..

[20] Zhang Z., Hu Y., Ji H., Lin Q., Li X., Sang S., McClements D.J., Chen L., Long J., Jiao A. (2023). Physicochemical stability, antioxidant activity, and antimicrobial activity of quercetin-loaded zein nanoparticles coated with dextrin-modified anionic polysaccharides. Food Chem..

[21] Zhang Z., Li X., Sang S., Julian McClements D., Chen L., Long J., Jiao A., Jin Z., Qiu C. (2023). Preparation, properties and interaction of curcumin loaded zein/HP-β-CD nanoparticles based on electrostatic interactions by antisolvent co-precipitation. Food Chem..

[22] Koirala P., Sablani S.S., Nirmal N. (2025). Gelatin/pectin based composite films re-enforced with mango peel extract loaded zein nanoparticles and zinc oxide nanoparticles: Enhanced mechanical and functional properties. LWT.

[23] Liu Q.R., Wang W., Qi J., Huang Q., Xiao J. (2019). Oregano essential oil loaded soybean polysaccharide films: Effect of Pickering type immobilization on physical and antimicrobial properties. Food Hydrocoll..

[24] Qiao D., Li M., Chen J., Lin L., Lu J., Zhao G., Zhang B., Xie F. (2025). Combination of crosslinked zein film enhances the water barrier and mechanical properties of deacetylated konjac glucomannan/agar-based bilayer films. Food Chem..

[25] Chen J., Zhou W., Liu G., Zhang H., Qin X. (2024). Effect of inulin with different degrees of polymerization on oxidation stability of whey isolate protein emulsion. Food Ferment. Ind..

[26] López-Castejón M.L., Bengoechea C., Espinosa S., Carrera C. (2019). Characterization of prebiotic emulsions stabilized by inulin and β-lactoglobulin. Food Hydrocoll..

[27] Zhu C.Y., Li K., Wang Y., Du M.T., Chen B., Wang Y.T., Zhou Y.F., Bai Y.H. (2025). Antioxidant and antimicrobial PSE-like chicken protein isolate films loaded with oregano essential oil nanoemulsion for pork preservation. Food Chem..

[28] Shao P., Zhang H., Niu B., Jin W. (2018). Physical stabilities of taro starch nanoparticles stabilized Pickering emulsions and the potential application of encapsulated tea polyphenols. Int. J. Biol. Macromol..

[29] Wang N., Liu X., Ma Y., Huang X., Song L., Guo H., Sun X., Sun X., Hai D., Zhao P. (2024). Identification of polyphenol extracts from flaxseed and study on its bacteriostatic mechanism. Food Biosci..

[30] Comaposada J., Marcos B., Bou R., Gou P. (2018). Influence of surfactants and proteins on the properties of wet edible calcium alginate meat coatings. Food Res. Int..

[31] Riaz A., Lagnika C., Luo H., Nie M., Dai Z., Liu C., Abdin M., Hashim M.M., Li D., Song J. (2020). Effect of Chinese chives (Allium tuberosum) addition to carboxymethyl cellulose based food packaging films. Carbohydr. Polym..

[32] Khan A., Riahi Z., Kim J.T., Rhim J.W. (2024). Chitosan/gelatin-based multifunctional films integrated with sulfur-functionalized chitin for active packaging applications. Food Hydrocoll..

[33] Tang J., Huang C., Liu W., Zeng X., Zhang J., Liu W., Pang J., Wu C. (2025). Preparation and characterization of a konjac glucomannan-based bio-nanocomposite film and its application in cherry tomato preservation. Food Hydrocoll..

[34] Giteru S.G., Coorey R., Bertolatti D., Watkin E., Johnson S., Fang Z. (2015). Physicochemical and antimicrobial properties of citral and quercetin incorporated kafirin-based bioactive films. Food Chem..

[35] Gong W., Yang T., He W., Li Y., Hu J. (2025). On-demand removable hydrogel film derived from gallic acid-phycocyanin and polyvinyl alcohol for fruit preservation. Food Chem..

[36] Kalkan S., Otağ M.R., Engin M.S. (2020). Physicochemical and bioactive properties of edible methylcellulose films containing *Rheum ribes* L. extract. Food Chem..

[37] Xiao H., Fu X., Cao C., Li C., Chen C., Huang Q. (2019). Sulfated modification, characterization, antioxidant and hypoglycemic activities of polysaccharides from *Sargassum pallidum*. Int. J. Biol. Macromol..

[38] Tan M., Zhong X., Xue H., Cao Y., Tan G., Li K. (2024). Polysaccharides from pineapple peel: Structural characterization, film-forming properties and its effect on strawberry preservation. Int. J. Biol. Macromol..

[39] Hou C., Wei H., Liu C., Yang H., Chen L., Xu J., Wang L., Rao X., Huang J., Ge Q. (2025). Application potential of submicron-sized bamboo cellulose as natural Pickering emulsion stabilizers: Structural properties and stabilization mechanisms. Food Chem..

[40] Fan B., Cui N., Xu Z. (2022). Thermoresponsive and selfhealing hydrogel based on chitosan derivatives and Polyoxometalate as an antibacterial coating. Biomacromolecules.

[41] Liu Z., Lin D., Shen R., Yang X. (2020). Characterizations of novel konjac glucomannan emulsion films incorporated with high internal phase Pickering emulsions. Food Hydrocoll..

[42] Shi S., Tang J., Zhou Y., Huang H., Zhang J., Liu W., Hu Z., Wang H., Pang J., Wu C. (2025). Development of sodium alginate-based active packaging films reinforced with curcumin/β-cyclodextrin stabilized high internal phase Pickering emulsions for blueberry preservation. Int. J. Biol. Macromol..

[43] Wang D., Du L., Sun Z., Liu F., Zhang D., Wang D. (2023). Characterisation, slow-release, and antibacterial properties of carboxymethyl chitosan/inulin hydrogel film loaded with novel antilisterial durancin GL. Carbohydr. Polym..

[44] Liu X., Xue F., Li C., Adhikari B. (2022). Physicochemical properties of films produced using nanoemulsions stabilized by carboxymethyl chitosan-peptide conjugates and application in blueberry preservation. Int. J. Biol. Macromol..

[45] Zhang J., Wang S., Wang L., Chen H., Wang Z., Hu Y., Jin J., Wu H., Liu Q. (2025). A pullulan/sodium carboxymethyl cellulose film incorporated with cinnamaldehyde-loaded nano-emulsions: Preparation, characterization, and application in blueberry preservation. Int. J. Biol. Macromol..

[46] Huang X., Li J., He J., Luo J., Cai J., Wei J., Li P., Zhong H. (2024). Preparation of curcumin-loaded chitosan/polyvinyl alcohol intelligent active films for food packaging and freshness monitoring. Int J. Biol. Macromol..

[47] Chen Q., You N., Liang C., Xu Y., Wang F., Zhang B., Zhang P. (2023). Effect of cellulose nanocrystals-loaded ginger essential oil emulsions on the physicochemical properties of mung bean starch composite film. Ind. Crop Prod..

[48] Kang S., Wang H., Xia L., Chen M., Li L., Cheng J., Li X., Jiang S. (2020). Colorimetric film based on polyvinyl alcohol/okra mucilage polysaccharide incorporated with rose anthocyanins for shrimp freshness monitoring. Carbohydr. Polym..

[49] Tang J., Zhang J., Si S., Huang H., Zeng X., Li Y., Liu W., Pang J., Wu C. (2025). Enhancing the antibacterial and antioxidant activities of chitosan/sodium alginate double-layer film by Pickering emulsion containing tea tree essential oil for food preservation. Food Chem..

[50] Hou Y., Sun Y., Jia S., Su W., Cheng S., Tan M., Wang H. (2024). Double-layer films based on konjac gum and hydroxypropyl methyl cellulose loaded with thyme essential oil Pickering emulsion: Preparation, characterization, and application. Food Hydrocoll..

[51] Zabihollahi N., Alizadeh A., Almasi H., Hanifian S., Hamishekar H. (2020). Development and characterization of carboxymethyl cellulose based probiotic nanocomposite film containing cellulose nanofiber and inulin for chicken fillet shelf life extension. Int. J. Biol. Macromol..

[52] Li H., Li M., Xu Y., Gao Z., Chi H., Wang H. (2025). Characterization of excellent performance tea polyphenol-loaded polyethylene oxide/ethyl cellulose nanofiber films prepared by centrifugal spinning and application in blueberry preservation. Food Biosci..

[53] Zhang D.Y., Yang J.X., Liu E.J., Hu R.Z., Yao X.H., Chen T., Zhao W.G., Liu L., Fu Y.J. (2022). Soft and elastic silver nanoparticle-cellulose sponge as fresh-keeping packaging to protect strawberries from physical damage and microbial invasion. Int. J. Biol. Macromol..

[54] Xu Y., Chen H., Zhang L., Xu Y. (2023). Clove essential oil loaded chitosan nanocapsules on quality and shelf-life of blueberries. Int. J. Biol. Macromol..

[55] Gu X., Li J., Yang L., Liu L., Xiao L. (2023). Preparation of natural complex waxy structure for the evaluation of preservation performance of blueberry. Food Biosci..

