# Supplementary material for: Chitosan-Based Composite Films Reinforced with Zein–Inulin–Thyme Essential Oil Pickering Emulsion for Enhanced Structural Integrity and Preservation Capacity
Source: Foods. 2026 Jan 31;15(3):484. doi: 10.3390/foods15030484 (PMC12896857; doi:10.3390/foods15030484)
Supplement: Supplementary file 1 [file foods-15-00484-s001.zip › foods-4089401-supplementary.pdf]

Supplementary material for:

Chitosan-based composite films reinforced with zein-inulin-thyme essential oil Pickering emulsion for enhanced structural integrity and preservation capacity

Liufeng Wang, Hongxin Xue, Yujie Ling, Xinping Zhong, Kuntai Li*, Qiuming Zheng, Xiaoqing Chen, Xinyi He and Minghui Tan*

College of Food Science and Technology, Guangdong Ocean University, Guangdong Provincial Key Laboratory of Aquatic Product Processing and Safety, Guangdong Provincial Engineering Technology Research Center of Seafood, Guangdong Province Engineering Laboratory for Marine Biological Products, Key Laboratory of Advanced Processing of Aquatic Product of Guangdong Higher Education Institution, Zhanjiang 524088, China

*Correspondence: atai78@sina.com (K.L.); tanminghui1020@163.com (M.T.)

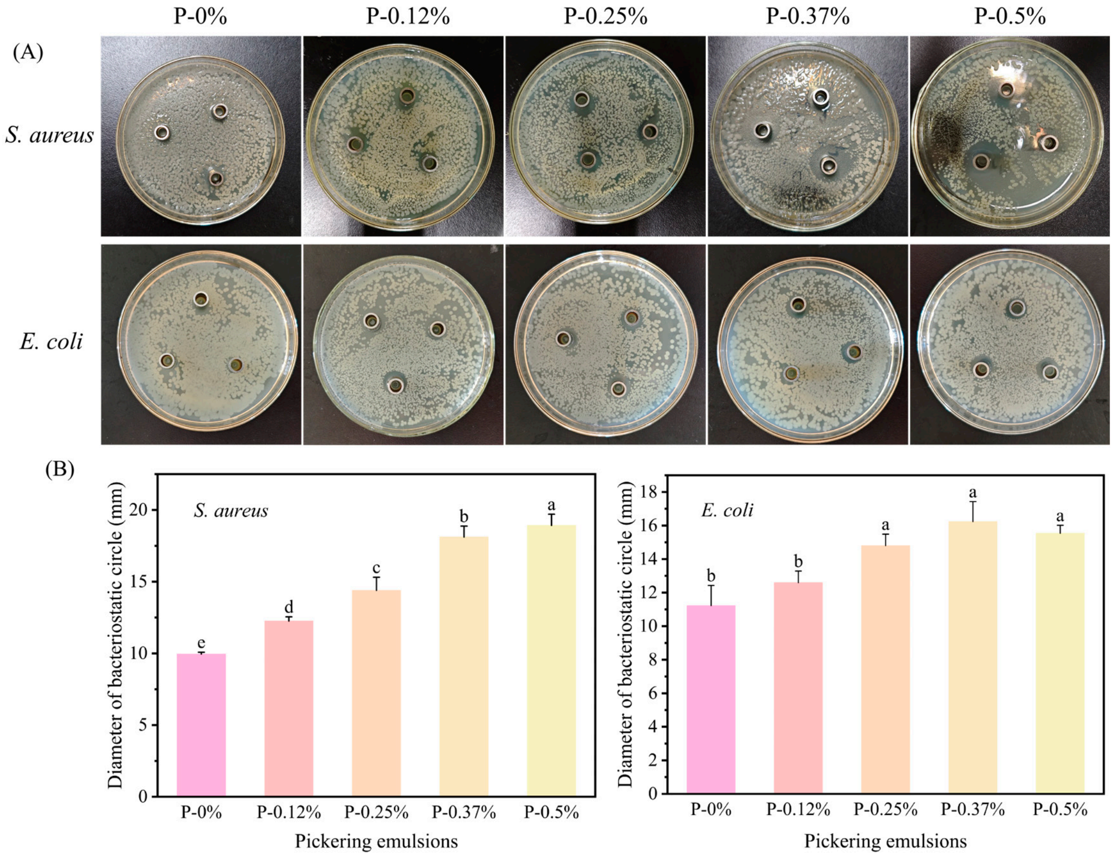

Figure S1. Antibacterial effect of Pickering emulsions. Distinct letters indicate significant differences ($P < 0.05$).
